# Assessment of men involvement in family planning services use and associated factors in rural Ghana

**DOI:** 10.1186/s13690-022-00822-5

**Published:** 2022-02-21

**Authors:** Senanu Abigail Kpekpo Kwawukume, Alexander Suuk Laar, Tanko Abdulai

**Affiliations:** 1 Seventh-Day Adventist Hospital, Koforidua, Eastern Region Ghana; 2 REJ Institute, Research and ICT Consultancy Services, Tamale, Northern Region Ghana; 3 Department of Community Health and Family Medicine, SMHS University for Development Studies, Tamale, Northern Region Ghana

**Keywords:** Family planning services, Men, Partners, Upper west region, Ghana

## Abstract

**Background:**

In low-and-middle income countries (LMICs) less attention is paid to men’s involvement in Family Planning (FP) programs where public health officials have advocated the involvement of men as a strategy for addressing the dismal performance of FP programs. The study assessed the involvement of men in FP use and the factors which promote or hinder the uptake of FP services among partners in a rural setting of northern Ghana.

**Methods:**

A cross-sectional descriptive study was used to collect data from 200 respondents. Study respondents were selected through random cluster sampling.

**Results:**

The findings showed that male partners’ knowledge (95.5%) and approval (72.8%) of FP services were high. About 48% of men were involved in FP service utilization. Having living children (aOR; 1.71(1.27, 2.15)) and being knowledgeable (aOR; 6.14(1.38, 10.90)) about FP were positively associated men’s involvement in FP service utilization. The findings also indicated that men had a higher propensity (X^2^ = 4.5534, *p* = 0.033) of supporting a FP method use. Women who reported that their spouse supported FP method use were more likely to use a contraceptive method (X^2^ = 9.5223, *P* = 0.002) if their spouse supported FP method use (X2 = 9.5223, *P* = 0.002) and if their partners had some education (X2 = 14.1133, *P* = 0.000). Reasons for low contraceptive use were health risks, side effects, and socio-cultural norms.

**Conclusion:**

Family planning programs need to include men at all levels of health promotion and education of FP programs to help reduce misconceptions about contraceptive methods to increase acceptance and use among partners in rural settings of Ghana.

## Background

Worldwide, 12% of married or in-union women are estimated to have had unmet need for contraceptive methods [1]. In LMICs, 214 million women of reproductive age who want to avoid pregnancy are not using modern contraceptive method [1]. In Sub-Saharan Africa, the proportion of women who have an unmet need for modern contraception is highest at 21% [1]. Unmet need for modern contraception and family planning (FP) accounts for 80% of unintended pregnancy in LMICs [1]. In Ghana, the contraceptive prevalence rate among all women aged 15–49 years is 25%, with 20% using modern methods [2] with a high unmet need of 45.7% [3]. Contraceptive use interventions and unmet need for FP are important determinants of fertility decline in LMICs [4]. Promotion of FP and ensuring access to preferred contraceptive methods for women and couples is essential to securing the well-being and autonomy of women [1]. Men participation in FP can be either as a user of male contraceptive methods and or encouraging and supporting their partners or wives in contraception [5]. Family planning services are critical to improving maternal and child health and reducing maternal and infant mortality in LMICs [6]. Expanding FP services and involving men in LMICs such as Ghana could increase utilization of services in this setting could avert up to 42% of maternal deaths [6, 7].

Men are also recognized to be responsible for the large proportion of reproductive ill-health suffered by their partners’ use of FP methods [8]. Although contraceptive methods and services are frequently geared towards women, men are often the primary decision-makers on family size and their partners’ use of FP methods [9]. It is well documented that men’s general knowledge and attitude about the ideal family size, gender preference of children, ideal spacing between childbirths, and contraceptive methods use greatly influence women’s preferences and opinions [8, 10, 11].

In Ghana, the focus of this study, knowledge of any contraceptive method is almost universal, with 98% of all women and 99% of all men knowing at least one method of contraception and where 50% of all women reported having used a method of contraception before [12]. According to the Ghana Demographic and Health Survey (2014), only 27% of married women use FP with 22% using a modern method and 5% using the traditional method [13]. Family planning programs in Ghana dates as far back as 1956 [14], however, uptake of FP services has not been encouraging and even worst in rural Ghana including the Sissala East District in Tumu of the Upper West Region where utilization of PF decreased from 71.4% in 2011 to 50.7% in 2012 [15]. In Ghana, The male partner may have an influence in decision-making regarding contraceptive use and the number of offspring they would like to have. Family planning research in rural Upper West Region has been dominated by findings almost exclusively from women studies.

Men in rural Ghana are seen to be the head of the home and influence the healthcare decisions of the entire household affairs [16]. Studies have also shown an increase in contraceptive use in cases where men partners have been involved [9, 17]. Men’s involvement helps not only in accepting contraceptives uptake but also its effective use and continuation [9, 10, 17]. However, male partners’ role in FP services promotion and uptake has often been overlooked and neglected in rural areas in Africa such as Ghana. To fill this research gap, this study assessed the involvement of men in FP service utilization and the factors that determine their involvement in contraceptive uptake among women in a rural district of the Upper West Region.

## Method

### Study setting

The study took place in Tumu in the Sissala East District in the north-eastern part of the Upper West Region of Ghana. Tumu, the district capital is predominantly rural by nature, with the majority (85%) living in rural settings [18, 19]. A large percentage (84%) of the population lives below the poverty line [18, 19]. A greater proportion (76%) of the population are being engaged in agriculture. It is predominantly Islam (88.0%) with Christianity being the largest of the minority (10%) followed by Traditional (1.4%) [18, 19]. A greater proportion of the population (52.4%) has Some level of education [18].

### Study design

A cross-sectional descriptive study design was used for this study. An interviewer-administered questionnaire consisting of both open and close-ended questions was administered by experienced research assistants to elicit the necessary information from the study population.

### Sampling technique and sample size

The sample size for this study was determined using the Yamane method: $\mathrm{Sample}\ \mathrm{Size}=\frac{\mathrm{N}}{1+\mathrm{N}{\left(\mathrm{p}\right)}^2}$ where *N* = Total Population, *p* = margin of error (5%). A sample size of 386 was derived from the total population of 11,252.

### Study population

The study population consisted of adult males and females aged 15 years and above who were either married or cohabitating. For this study, we aimed at recruiting 386 respondents but finally recruited 200 respondents due to logistical constraints.

### Data collection method

An interviewer-administered questionnaire consisting of both closed and open-ended questions was used to assess the knowledge and use of FP services among partners and the level of involvement of male partners in FP service utilization. The study questionnaire was developed based on the objective of this study by the principal investigator. The questionnaire was administered by the first author and two experienced research assistants who understand the native dialect. The questionnaire was translated into the local dialect and administered by the two research assistants for participants who could not understand or speak the English language. The questionnaire was administered among households in five communities or clusters out of the ten in Tumu municipality. These communities were chosen using a simple random sampling method. Forty respondents were interviewed from each community. This method gave the individual an equal chance of being selected. Before consent was sought from respondents, the aim of the study was explained to each individual. They were also assured of confidentiality and privacy of the information they will give. The questionnaires were pretested in a similar environment in the district.

### Outcome variable

Male participants were asked whether they approved and/or encourage use FP of their partners, ever discussed FP with their partners, provided material support to their partners to access FP services and whether they have used FP themselves in the past. Responses to these questions were them summed to give the level of male partners’ involvement in FP utilization (with no weight given to these variables); the highest possible involvement score was five (indicating a high level of involvement), a zero score indicated no involvement in FP utilization, and score of 3 was considered as sufficient involvement. The involvement of men in FP service utilization for themselves and/or their partners was the main outcome of the study. Female respondents were also asked; if they discuss FP with their male partners, gets approval to use FP from their male partners and whether they receive support from their male partners to access FP services.

### Covariates

The demographic characteristics (age, education, occupation, and religion) and knowledge about FP were the covariates considered for our analyses.

### Data management and analysis

To ensure accuracy, the data collected was checked and screened for completeness. The completed copies of the questionnaire were serially numbered and doubly entered and analyzed using Statistical Package for Social Scientists version 20.0. Bivariate and regression analyses were used to determine the associations between the outcome variables and a host of explanatory variables.

## Results

### Background characteristics of respondents

Two hundred (200) respondents were interviewed for this study. The study involved 107 men representing 53.5% and 93 women representing 46.6%. Respondents’ age ranged between 15 and 54 for men and 15–49 for women. In all, over 70 % (76.5%) had some form of education ranging from primary to tertiary while 23.5% had no education (Table 1). All the respondents were involved in some kind of work with over 60 % (66%) being artisans.

**Table 1:** Background characteristics of participants

	Male (*n* = 107)		Female (*n* = 93)	
	Frequency	Percent (%)	Frequency	Percent (%)
Age				
15–24	25	23.36	47	50.54
25–34	40	37.38	19	20.43
35–44	19	17.76	19	20.43
45–54	8	7.48	8	8.60
55+	15	14.02		
Marital Status				
Single	43	40.57	36	38.71
Married	56	52.83	52	55.92
Cohabiting	6	5.66	3	3.23
Divorced/widowed	1	0.94		
*Number years in current relation*				
< 5 years	44	52.38	56	66.67
5–9 years	15	17.86	9	10.71
10+ years	25	29.77	19	22.62
*Education*				
None	17	15.89	30	32.26
Primary	5	4.67	7	7.53
Junior High School	16	14.95	19	20.43
High School	26	24.30	27	29.03
Tertiary	43	40.19	10	10.75
*Occupation*				
Farming	7	8.24	16	17.20
Trading	22	25.88	22	23.66
Teachings	16	18.82	3	3.23
Health worker	6	7.06	5	5.38
Others	34	40.00	47	50.54
*Religion*				
Christianity	41	38.32	24	25.81
Islam	63	58.88	69	74.19
Traditional	2	1.87		
Other	1	0.93		
*Number of living children*				
0	54	50.46	36	38.71
1	15	14.02	20	21.51
2	11	10.28	16	17.20
3	10	9.35	10	10.75
4	10	9.35	5	5.38
5	4	3.74	4	4.30
> 5	3	2.80	2	2.15
*Desired number of children*				
1	2	1.87	1	1.08
3	31	28.97	21	22.58
4	32	29.91	31	33.33
5	11	10.28	15	16.13
> 5	17	15.89	11	11.83
Whatever God gives	9	8.41	9	9.68
*Ideal birth interval (years*)				
1	1	0.93	11	11.83
2	20	18.69	45	48.39
3	57	53.27	3	3.23
Don’t know	29	27.10	34	36.56
*Knowledge of FP*	78	76.47	80	86.96
*Approves partner’s use of FP/partner approves use of FP*	75	72.82	69	75.00
*Use FP*	48	52.17	32	36.36
*Discusses FP with partner*	58	65.10	59	64.13
*Ever asked partner to use FP*	38	41.76		
*Provide support for partner to use FP/receives support from partner*	46	52.27	59	67.05
*Involvement score*				
0	16	14.95		
1	22	20.56		
2	17	15.89		
3	21	19.63		
4	9	8.41		
5	22	20.56		
*Sufficient FP involvement*	52	48.60		

### Knowledge of family planning

Majority of the male respondents (95.5%) had heard of FP. Almost half of the respondents (48.1%) had information about FP via the mass media (Television, Radio, and Newspaper) followed by friends (27.5%) and the health facility (23.5%) as indicated in Table 2.

**Table 2:** Source of Information on family planning (male respondents)

Source	Frequency	Percent (%)
Television	17	16.7
Radio	30	29.4
Newspaper	2	2.0
Internet	1	0.9
Friends	28	27.5
Health facility	24	23.5
**Total**	**102**	**100.0**

Twenty eight percent (28.0%) of the men interviewed understood FP as avoidance of unintended pregnancy, 25.2% as limiting family size while 19.6% understood it as spacing of childbirth. Others (27.1%) explained it as two or more of the above definitions as shown in Table 3. The most common method known and used was the condom (42.5%) followed by implants (32.0%) and the least known method being the foaming tablet (7.0%) among male respondents.

**Table 3:** Men understanding of meaning of family planning

Meaning	Frequency	Percent (%)
Limiting family size	27	25.2
To avoid unintended pregnancy	30	28.0
Spacing childbirth	21	19.6
Others	29	27.1
**Total**	**107**	**99.9**

### Men involvement in FP

Over 50 % (52.2%) of the male respondents reported they or their partner were currently using some form of contraceptives to delay or avoid pregnancy. However, only 36.4% of women reported they or their partners were currently using contraceptives to delay or avoid pregnancy. The majority of the men respondents (72.8%) approved of the use of FP methods by their partners, 75% of the women respondents also indicated their partners had approved of their use of FP. The findings also indicate that men had a higher propensity of reporting FP use (X^2^ = 4.5534, *p* = 0.033). For the couples who did not approve of the FP methods, the reasons included: socio-cultural beliefs (31%), side effects (30.8%) such as delayed or absence of menses, and difficulty of conceiving after terminating use of FP and others (38.5%).

Overall, about 48% of men were sufficiently involved in FP service utilization (see Table 1). Involvement of men in FP use was positively associated with knowledge of FP and the number of living children (Table 4).

**Table 4:** Determinants of male involvement in FP

	Unadjusted OR (CI)	***P***-value	Adjusted OR (CI)	***P***-value
Age	1.28(0.99, 1.57)	0.0556	1.08(0.74, 1.42)	0.7004
Marital Status	1.13(0.84, 1.42)	0.4215	0.91(0.70, 1.12)	0.4743
Number of years in relationship	0.92(0.65, 1.19)	0.6479	0.77(0.48, 1.06)	0.2657
Education	1.17(0.92, 1.42)	0.1983	1.19(0.95, 1.43)	0.1003
Occupation	1.21(0.97, 1.45)	0.0973	1.28(1.00, 1.56)	0.0508
Religion	0.57(0.13, 1.01)	0.0550	0.66 (0.16, 1.16)	0.1432
**# of living children**	**1.32(1.11, 1.53)**	**0.0023**	**1.71(1.27, 2.15)**	**0.0003**
Desired # of Children	1.02(0.81, 1.23)	0.8848	0.93(0.71, 1.15)	0.5600
Desired birth intervals	1.34(0.98, 1.70)	0.0675	1.30(0.95, 1.65)	0.0903
**FP Knowledge**	**5.81(1.24, 10.38)**	**0.0256**	**6.14(1.38, 10.90)**	**0.0179**

# number, *FP* Family Planning. Age, education, Religion, and occupation were adjusted for

### Decision making in FP use

**Fig. 1:**
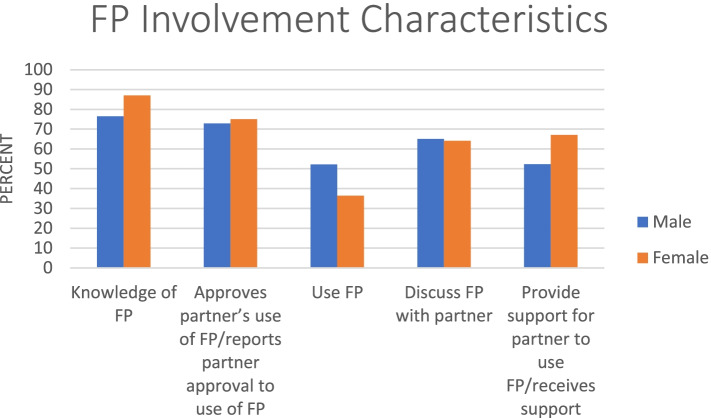
FP involvement characteristics

We wanted to know from the women whether their partners support them in their desire to use contraceptives; 67.0% of the women answered in the affirmative and 33.0% answered in a negative. Those who answered in the affirmative said their partners support them by providing money for transport to facility and/or for FP services, encouraging and accompanying them to the health facility. Women who reported that their spouse support FP use were more likely to use a contraceptive method (X^2^ = 9.5223, *P* = 0.002) compared to those who said no. Women who reported their partners had some education were also more likely to use a contraceptive method (X^2^ = 14.1133, *P* = 0.000). In all, female respondents tended to report more favorable attributes for their male partners’ involvement than the male respondents (Fig. 1).

Generally, a greater proportion of men (77.6%) intend using FP in the future. For those who answered in a negative, 19.1% said most of the contraceptives were designed to suit women and thought it was a woman’s business. Nearly three quarters of women (73.4%) who ever used a FP method indicated their partner had a say in the decision to use. For the women interviewed,70.7% said they would still practice birth control irrespective of their partners’ opinion while 23.9% would not use birth control if their partners were against it, 5.4% were however not certain.

## Discussion

This study assessed male partners’ involvement and factors associated with the use of FP services in Tumu in the Sissala East District of the Upper West Region of Ghana. The study identified several factors that influence FP services use among male partners in this setting. Our study demonstrated that despite the high knowledge of modern contraceptive methods among couples, use was low due to perceived side effects and socio-cultural beliefs. Men’s attitude and social practice towards FP methods also influence the behavior of their partners using contraceptives [20]. It is found elsewhere that the decision not to practice FP is men-dominated and men are responsible for providing contraceptive decisions when FP is practiced [21]. A major limitation facing low-and-middle income countries FP promotion programs and population policy development on contraceptive behavior is that men are often not targeted in FP programs [22].

Our study identified several factors associated with men’s influence in FP service utilization among their partners. Non-approval of  FP methods by men in this study was attributed to perceived risks, side effects, and socio-cultural norms. Focus group discussions with men and women in rural Uganda have come out with similar findings [9]. Contraceptive knowledge and use are shaped by the socio-cultural environment such as personal attitudes and feelings about contraception. In rural settings in low and -middle income countries most men may be unwilling to have their wives adopt FP, which they have little knowledge about. Evidence shows that some men oppose contraceptive use for reasons of tradition and religion which require men to maintain the honor and position of their extended family, village, religious group and social organization [23]. Studies have shown with similar findings in settings in rural northern Ghana [24, 25]. The complex web of social and cultural factors impedes spousal communication regarding reproductive health issues and that discourages them to take their wives to health clinics to discuss FP issues [22].

Two important factors were identified to be positively associated with men’s involvement in FP use in our study; Knowledge on FP and the number of living children male partners had were positively associated with their involvement in FP use. Knowledge about FP will influence acceptance and therefore impact involvement of men in its utilization. Similar studies by [26, 27] have found FP knowledge to be positively linked with its utilization. Our study additionally revealed that men who had greater than two living children were more likely to be involved in FP service utilization. The number of living children have also been shown to be associated with contraceptive use among women in previous studies [28, 29]; couples with living children tend to use contraceptives space their births or limit the number of children. Several interventions can be used to address barriers in the uptake of FP services in this setting. Family planning programs need to target men at all levels of health promotion and education with their partners to reduce misconceptions about FP methods to increase acceptance [20]. Men’s participation is crucial to help reduce misconception about side effects of contraceptive methods [20]. Therefore, FP family programs need to target men at all levels of the service. Their involvement will also lead to women’s empowerment to increase effective contraceptive use and continuation to improve better health outcomes in reproductive health [30]. User experiences indicate that text messages provide a novel way to raise awareness, promote behavior change and address myths and socio-cultural norms [31].

### Limitations

This study has some limitations which need to be taken into consideration. The finding of this study cannot be generalized to the entire region of the Upper West region due to the small sample size. Despite the small sample size, views of groups of our respondents which comprised of married partners and those cohabitating, will not differ significantly from the rest of the entire population in the region. Also, this study provides vital insights for policymakers in Ghana and beyond who are working to improve sexual and reproductive health services for men and women. The need for future study to capture the perspectives of men and women on cultural factors influencing PF services for policy.

## Conclusions

Our study demonstrated high knowledge of FP among partners. However, the use of modern contraceptives methods was low due to side effects and socio-cultural norms. Involving men partners in FP programs could give them accurate and complete information on contraceptive methods to help reduce misconception and increase uptake. Reproductive health program designers, policymakers, and population researchers, health professionals need to incorporate the findings into reproductive health programs to help address barriers to improve health outcomes among couples.

## Data Availability

The dataset for this study is available on request from the corresponding author.

## Abbreviations

FP: Family Planning
LMICs: Low- and Middle-Income Countries
